# The end of the reign of a “master regulator’’? A defect in function of the LasR quorum sensing regulator is a common feature of *Pseudomonas aeruginosa* isolates

**DOI:** 10.1128/mbio.02376-23

**Published:** 2024-02-05

**Authors:** Mylène C. Trottier, Thays de Oliveira Pereira, Marie-Christine Groleau, Lucas R. Hoffman, Ajai A. Dandekar, Eric Déziel

**Affiliations:** 1 Centre Armand-Frappier Santé Biotechnologie, Institut National de la Recherche Scientifique (INRS), Laval, Québec, Canada; 2 Departments of Pediatrics and Microbiology, University of Washington, Seattle, Washington, USA,; 3 Departments of Medicine and Microbiology, University of Washington, Seattle, Washington, USA; Georgia Institute of Technology, Atlanta, Georgia, USA

**Keywords:** quorum sensing, *Pseudomonas aeruginosa*, virulence regulation, ecology, adaptive mutations, opportunistic infections

## Abstract

*Pseudomonas aeruginosa*, a bacterium causing infections in immunocompromised individuals, regulates several of its virulence functions using three interlinked quorum sensing (QS) systems (*las*, *rhl*, and *pqs*). Despite its presumed importance in regulating virulence, dysfunction of the *las* system regulator LasR occurs frequently in strains isolated from various environments, including clinical infections. This newfound abundance of LasR-defective strains calls into question existing hypotheses regarding their selection. Indeed, current assumptions concerning factors driving the emergence of LasR-deficient isolates and the role of LasR in the QS hierarchy must be reconsidered. Here, we propose that LasR is not the primary master regulator of QS in all *P. aeruginosa* genetic backgrounds, even though it remains ecologically significant. We also revisit and complement current knowledge on the ecology of LasR-dependent QS in *P. aeruginosa*, discuss the hypotheses explaining the putative adaptive benefits of selecting against LasR function, and consider the implications of this renewed understanding.

## PERSPECTIVE

### Current understanding of *Pseudomonas aeruginosa* quorum sensing

The bacterium *Pseudomonas aeruginosa* is a versatile opportunistic pathogen found mostly in environments related to human activity such as soil, water, and hospital settings (1). *P. aeruginosa* causes infections in immunocompromised individuals and people living with cystic fibrosis (CF) (2–4). To adapt to diverse and dynamic environments, this bacterium uses quorum sensing (QS), an intercellular communication system. Through the production and detection of small diffusible autoinducers, QS coordinates the expression of numerous key cellular functions at the population level, including virulence, in a cell density-dependent manner (5).

*P. aeruginosa* has three interdependent QS systems, known as *las*, *rhl*, and *pqs*. Based on observations of prototypical strains such as PA14 and PAO1*,* the *las* system is generally considered to be at the top of the QS regulatory cascade (6, 7). This system comprises the LasI synthase, which produces the autoinducer *N*-(3-oxododecanoyl)-L-homoserine lactone (3-oxo-C_12_-HSL). This signal molecule subsequently binds to its cognate LuxR-type transcriptional regulator, LasR. Once activated by its ligand, LasR regulates the transcription of several target genes, such as *lasB* encoding for the LasB elastase, as well as *lasI*, which codes for the LasI synthase, completing a positive feedback loop that is a characteristic of many QS systems (8–10). LasR activation also induces the transcription of the *rhlI* and *rhlR* genes, which constitute the *rhl* QS system (6, 7, 10–12). The RhlI synthase produces the *N*-butanoyl-L-homoserine lactone (C_4_-HSL) autoinducer signal, which can bind to another LuxR-type transcriptional regulator, RhlR, activating the transcription of several genes, including those implicated in the production of virulence factors, including pyocyanin (*phz1* and *phz2* operons) and rhamnolipids (*rhlAB* and *rhlC*) (11, 13–15). The third QS system, *pqs*, is regulated by MvfR (also known as PqsR), a LysR-type transcriptional regulator. The *pqs* system relies on the production of 4-hydroxy-2-alkylquinolines (HAQs) by the enzymes encoded by the *pqsABCDE* operon (16, 17). Indeed, the PqsABCD enzymes synthesize 4-hydroxy-2-heptylquinoline (HHQ), which can be converted into the *Pseudomonas* quinolone signal (PQS; 3,4-dihydroxy-2-heptylquinoline) by the genetically unlinked PqsH monooxygenase (16, 18, 19). HHQ and PQS can both act as autoinducing ligands of MvfR, leading to the transcriptional activation of the *pqs* operon (18, 20).

The interconnection of all three systems is widely recognized. LasR activation positively regulates the expressions of *rhlR*, *mvfR*, and *pqsH*, while RhlR activation represses *pqs* operon activity (10, 15, 17, 21–24). Additionally, PqsE, a thioesterase enzyme encoded by the last gene of the *pqs* operon, plays an important role in the regulation of *P. aeruginosa* QS (25). Indeed, this protein interacts with RhlR through an incompletely defined chaperone-like function to activate the *rhl* system and heighten the expression of some target genes (12, 26–29). These examples highlight the complex regulation that characterizes *P. aeruginosa* QS. However, while most of the research that informed this interconnected but hierarchical model of QS was performed in a few, well-defined laboratory strains, the frequent detection of LasR-defective strains still able to produce QS-regulated factors has revealed certain inconsistencies regarding the central role of the LasR regulator within the QS hierarchy. Here, we will propose an alternative perspective and discuss this issue based on current literature, unveiling new hypotheses regarding the relative positions of LasR, RhlR, and MvfR (PqsR) within the *P. aeruginosa* QS hierarchy.

### LasR-defective strains are generally prevalent in both chronic and acute clinical environments

Over time, numerous studies have highlighted the natural occurrence of *P. aeruginosa* strains with impaired LasR activity. Initial reports of LasR-defective variants reflected their detection within microbial populations that had evolved for years within the chronically infected lungs of people with CF, which was unexpected given the requirement of QS-controlled factors for full expression of bacterial virulence *in vitro* (30). However, multiple reports have identified LasR-defective strains in CF respiratory cultures, and the selection of these strains by the CF lung environment is now a well-accepted dogma. The emergence of *lasR* mutants has been associated with increased inflammatory markers, increased neutrophilic inflammation, and deteriorated pulmonary function (31–37). These variants have also been implicated in pathogenesis in corneal ulcers and in the airways of individuals with chronic obstructive pulmonary disease (COPD) and ventilator-associated pneumonia (38–40). Moreover, we recently identified a high prevalence of LasR-defective strains in non-clinical environments as well, such as sinks, hydrocarbon-contaminated soils, and animal products (4). While a defect in LasR activity has seldom been reported in strains isolated from acute infections, a recent study from O’Connor et al. (41) identified *P. aeruginosa* isolates carrying *lasR* mutations commonly present within many environments, although the impact of these mutations on LasR activity *per s*e was not investigated (38, 41, 42).

**Fig 1:**
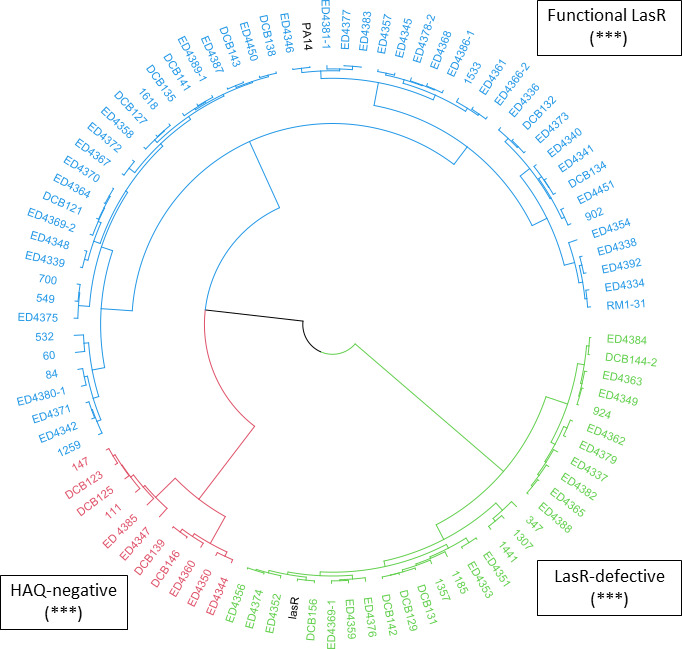
Clustering analysis performed on a panel of 92 strains of *P. aeruginosa* isolated from acute and chronic infections. Clustering analysis is based on selected variables from a previous study. Briefly, HAQ concentrations and pyocyanin production were measured in King’s A medium at two different time points (4). Strains PA14 (Functional LasR; top) and PA14 *lasR*::Gm (LasR-defective; bottom, “lasR”) were included in the analysis as references. Three robust clusters were identified. One of these clusters comprises LasR-defective strains, whereas another includes strains with a functional LasR protein. The third cluster comprises strains that produce negligible levels of HAQs. Further analyses are required to confirm the functionality of LasR in this subset of isolates, as described before (4). Raw data used to generate this analysis are presented in Table S2. Statistical analyses were made as previously described using R software (4, 44). Robustness (*) represents the proportion of clustering runs in which a pair of isolates appeared together in some clusters, given that they were clustered together in at least one run, averaged over all such pairs (***: 80%–89%).

Based on the emerging picture of a widespread prevalence of LasR-defective strains in various environmental niches, we hypothesized that defects in LasR activity could also be found in acute clinical contexts. To evaluate the prevalence of LasR-defective strains isolated from both chronic and acute infections, we assessed LasR activity in 92 *P*. *aeruginosa* strains from diverse sources, including burn wounds, keratitis, urinary tract infections, bronchitis, CF lungs, COPD, and others (Table S1) (43). LasR activity was evaluated using a previously published method relying on unbiased phenotypic profiling based on the quantification of QS-regulated metabolites such as HAQs and pyocyanin (4). Based on this model, we found that 41% of the 92 isolates from our panel of clinical strains exhibited impaired LasR activity (Fig. 1). These data are consistent with our previous findings, where 40% of isolates from a panel of 176 *P*. *aeruginosa* environmental strains were found to be LasR-defective (4). These results also support the conclusions of O’Connor et al., as well as our hypothesis that LasR-defective strains are common in all clinical contexts and are not restricted to CF clinical isolates, as previously assumed (41), due to a predominant focus of prior analyses on CF isolates. Here, we found the prevalence of defects in LasR function to be similar among isolates from various environments, including when comparing CF and non-CF sources.

In the literature, the prevalence of *lasR* mutants among *P. aeruginosa* isolates from CF patients has been estimated to be from 20% to 25%, as defined using genotypic techniques (35, 45, 46). However, different studies have used a variety of methods to estimate the proportion of LasR-defective strains, hampering direct comparison of results. Relying solely on the identification of mutations in the *lasR* coding sequence, as it is usually reported, could misidentify LasR-defective strains, given its complex regulation and the unpredictable relationship between coding sequence and protein function (4, 35, 47). Indeed, a great diversity of mutations in the *lasR* gene has been identified, not all of which completely abrogate function (35). Additionally, some mutations in regulatory elements could also modify LasR expression or activity (4, 35). Examining a single phenotypic trait could also bias LasR-defective strain identification given the complex regulation of many phenotypes. Our approach, based on phenotypic profiling rather than examining only one trait, allows for unbiased identification of all isolates with a defect in LasR activity (4). Therefore, we refer to LasR-defective strains rather than *lasR* mutants.

Taken together with previous studies, the data presented here further highlight that LasR-defective strains occur commonly, regardless of their clinical or environmental origin (4, 35). As a result, we must reconsider prior assumptions, including the notion that chronically colonized CF lungs distinctively provide a selective environment promoting the loss of LasR function (32, 33).

### Factors driving the emergence of LasR-defective strains remain elusive

Despite decades of research, the precise drivers for the emergence of LasR-defective strains remain elusive. LasR function seems especially prone to be lost, and the *lasR* gene might even be considered a hotspot for various types of genetic variations (4, 35, 41). One particularly popular hypothesis suggests that LasR-defective cells arise as “cheaters,” taking advantage of neighboring cells with a functional LasR that provide “public goods,” such as exoproteases (e.g., LasB elastase), to the entire population. By exploiting this strategy, LasR-defective variants reduce the population metabolic cost associated with the expression of these proteins when they are essential for bacterial growth. Interestingly, this behavior has been mostly observed under specific *in vitro* conditions (39, 48–51), as well as suggested under *in vivo* conditions (52, 53). While such social cheating behavior could potentially extend to different contexts and environments beyond the CF lung, providing an interesting explanation for the emergence of these variants in multiple niches, evidence suggests that social conflict is not the sole source of LasR loss-of-function and that *lasR* mutants are not always “cheaters” (54). It is important to consider that LasR-defective strains can also arise in various conditions that do not implicate “public goods” (55). Therefore, other factors must contribute to the emergence of LasR variants.

As stated above, LasR-defective strains have been typically associated with the chronically infected lungs of people with CF (30, 32, 56). Extensive investigations have also explored the non-social benefits conferred by defective LasR function in this specific environment, revealing their relatively high fitness in the presence of specific amino acids, such as phenylalanine, which is especially abundant in CF secretions (32, 57). Relative to wild-type strains, these variants exhibit altered metabolism, including lower oxygen consumption and enhanced nitrogen utilization, providing them with a competitive edge over their wild-type counterpart (56, 58). Furthermore, LasR-defective strains exhibit relative resistance to specific antimicrobials and enhanced tolerance to alkaline stress, resulting in protection from cell lysis (32, 33, 59–61). These findings support the notion that diminished LasR activity confers substantial advantages in conditions known to be common in CF lungs. However, the growing evidence for the prevalence and abundance of LasR-defective variants in diverse infections and environmental contexts disproves any specificity for the CF lung and warrants expanding current models of their emergence. For instance, it was proposed that diversification of *P. aeruginosa* populations in the CF lung could favor long-term survival to multiple unpredictable stresses (62). This phenomenon could also apply outside of the CF lung. Accordingly, the effects of LasR impairment on growth on different carbon sources were suggested to explain the emergence of LasR-defective variants in the context of the CF lung, but such non-social mechanisms could likely arise in many other environmental settings, as growth conditions and carbon sources differ (63).

Alternatively, we might consider the emergence of LasR-defective variants as beneficial for a population that contains them (64). Supporting this model, controlled evolution experiments performed *in vitro* demonstrated the tendency of LasR-defective clones to rapidly emerge, with their proportion frequently stabilizing at about 50% of the total population (48, 50, 55, 59, 65). For instance, swarming colonies of *P. aeruginosa* with higher proportions of LasR-defective cells tend to have fitness advantages over those without LasR variants (55). The population dynamics of mixed populations of *P. aeruginosa*, comprising wild-type and LasR-defective strains, could partially explain the prevalence of the latter in diverse environments. In natural settings, *P. aeruginosa* forms biofilms, which are bacterial communities composed of diverse physicochemical niches. Jeske et al. showed that LasR function is especially prone to be lost in a biofilm context (42). Within these biofilms, factors produced by strains with a functional LasR could influence the behavior of surrounding LasR-defective strains within specific niches. Similarly, LasR-defective strains can modulate the behavior of LasR-functional strains by affecting, for instance, QS signaling and function (66, 67).

Hence, the concomitant mixed presence of LasR-functional and LasR-defective strains appears to be beneficial to the overall population. This observation suggests that LasR plays an important ecological role and remains essential for the viability of *P. aeruginosa* populations. There could be various reasons for the selection of such mixed populations, including both social and non-social drivers, considering the multitude of factors that influence the diversification of this protein.

### LasR-defective clinical isolates could be acquired directly from surrounding environments

The same high prevalence of LasR-defective strains in both clinical and environmental contexts suggests potential adaptative benefits associated with modulating LasR activity in populations of *P. aeruginosa*, irrespective of the environment. Therefore, it is possible that LasR-defective strains isolated from infections originated from environmental sources, as previously proposed, rather than emerging *in situ* (4, 38). Two distinct studies provided evidence supporting this notion, as the *lasR* gene of some *P. aeruginosa* isolates already exhibited mutations at initial detection in CF respiratory samples, consistent with a direct acquisition from environmental sources (40, 68). While *P. aeruginosa* lineages have been observed to lose LasR function over time during an infection, the discussion above indicates that such events are not necessarily specifically selected by the CF lung environment (30, 69–72).

### Presence of naturally occurring LasR-impaired function is not necessarily synonymous with loss of quorum sensing

**Fig 2:**
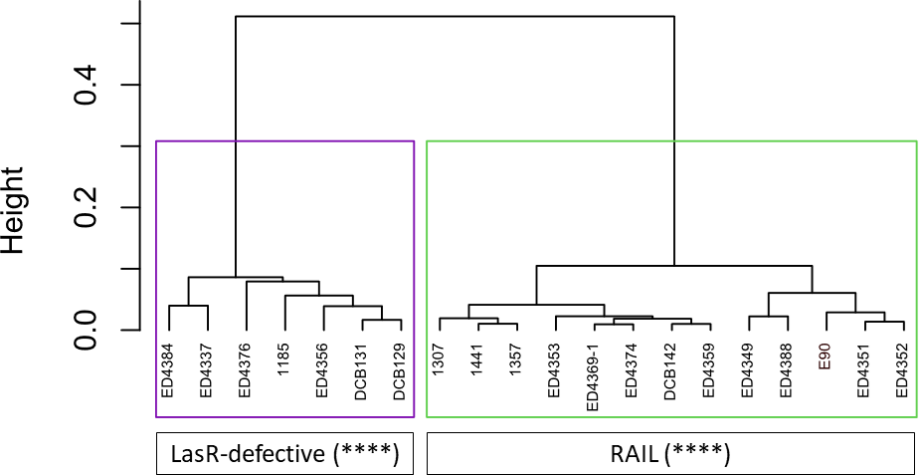
Clustering analysis performed on a subset of LasR-defective isolates of *Pseudomonas aeruginosa* shown in Fig. 1. Clustering analysis was based on chosen variables from a previous study. Briefly, HAQ concentrations, pyocyanin production, and activity of a *rhlA-gfp* reporter were measured in King’s A medium at two different time points using the same methods as previously described (4, 82). RhlR Active Independently of LasR (RAIL) strain E90 was included in the analysis as a reference. Raw data used to generate this analysis are presented in Table S2. Statistical analyses were made as previously described using R software (4, 44). Robustness (*) represents the proportion of clustering runs in which a pair of isolates appeared together in some clusters, given that they were clustered together in at least one run, averaged over all such pairs (****: >90%).

A widely held notion is that LasR occupies the top position in the QS regulatory hierarchy (47, 73). An alternative hypothesis is that the presence of this hierarchy is not a universal feature of *P. aeruginosa* and reflects studies conducted primarily with one strain, PAO1, which could itself be considered as an outlier regarding its QS mechanisms (74). We now know that isolates with defective LasR activity can still exhibit robust QS-dependent regulation of virulence factors (4, 35, 75–77). Since LasR upregulates the *rhl* and the *pq*s QS systems in studied laboratory-adapted strains, it has been generally assumed that a defect in LasR activity should result in deficient QS regulation and thus reduced transcription of target genes and production of virulence factors. However, LasR-defective strains with a functional *rhl* QS system, referred to as “RAIL’’ (RhlR Active Independently of LasR) strains, have been identified. Such strains have been isolated from CF lungs, as well as from diverse environments (4, 35, 75–77). Using the same method as previously mentioned, we found that about half of the LasR-defective clinical strains from our panel (Fig. 1) possess LasR-independent RhlR activity (Fig. 2), in accordance with previously published findings (4). Hence, an impaired LasR protein does not necessarily lead to diminished production of virulence factors. Thus, the dogma based on the study of prototypical strains stating that LasR is the conserved “master regulator” of the QS regulatory cascade should be reconsidered. Besides regulating known RhlR-dependent virulence factors such as pyocyanin, RhlR can activate virulence factors typically regulated by LasR, such as various exoproteases (4, 35, 78, 79). Together, these observations and concepts highlight the crucial role of RhlR, accentuating its importance as a QS regulator in *P. aeruginosa*. However, the precise regulatory mechanisms involved in LasR-independent RhlR regulation remain elusive. One mechanism might involve PqsE, encoded by the last gene of the *pqs* operon. PqsE plays a major role in promoting RhlR activity, at least in prototypical strains (12, 26, 29, 80, 81), underscoring the importance of MvfR and the *pqs* system for maintaining the full suite of QS regulation. Since an important subset of naturally evolved *P. aeruginosa* strains activates QS and produces virulence factors without relying on LasR, we need to better understand the nature of the ecological pressure sustaining its regulatory activity in some lineages.

### Improving quorum sensing inhibition by targeting RhlR-dependant factors

*P. aeruginosa* is naturally tolerant of, and resistant to, a wide range of antibiotics, limiting the efficacy of treating infections by this bacterium (83). To address this issue, anti-virulence therapies have emerged as a promising approach for the development of new drugs, since they offer distinct advantages. Unlike antibiotics that directly target the survival of the bacteria, anti-virulence therapies aim to inhibit non-essential virulence factors without affecting viability. This approach theoretically reduces the development of resistance since no selective pressure for survival is applied. In *P. aeruginosa*, QS represents an interesting target, since it modulates the expression of multiple virulence factors unrelated to bacterial survival (84–87). Rationally, many anti-virulence therapies target *P. aeruginosa* LasR or the production of 3-oxo-C_12_-HSL, since the *las* system has been considered to be on top of the QS regulation cascade (88–90). Unfortunately, the high prevalence of LasR-defective strains and the clear absence of QS hierarchy in some strains suggest that LasR is not an ideal target. Instead, we propose that researchers working on anti-QS therapies should consider focusing on other QS targets, such as the *rhl* or *pqs* system, which can still be active in the absence of LasR and seem better conserved. There have been a very few reports of strains lacking RhlR or MvfR/PqsR activity, perhaps because these regulators are indispensable for the proper functioning of QS (41, 91–93). Thus, targeting *rhl*-dependent QS, such as the production/function of C_4_-HSL or PqsE, could be an interesting approach in the control of *P. aeruginosa* infections and their clinical consequences. Studying the QS ecology in strains from a broader range of origins (clinical and environmental) should allow for better target selection.

### Outstanding questions and conclusion

Acknowledging that maybe 40% of all *P. aeruginosa* isolates are LasR-defective raises several important questions. One particularly intriguing observation is the prevalence of mutations in *lasR* compared to those in its cognate synthase gene, *lasI* (41). A possible explanation for this discrepancy could be the absence of social benefit for *lasI* mutations: the inactivation of LasI would not prevent an autologous response to neighboring strains producing the diffusible 3-oxo-C_12_-HSL signal in a mixed population (63). Another potential explanation is that 3-oxo-C_12_-HSL does not exclusively serve as a LasR autoinducer and contributes to alternative functions in *P. aeruginosa*. For example, 3-oxo-C_12_-HSL increases pyocyanin production in the absence of LasR (67). In fact, the maintenance of 3-oxo-C_12_-HSL production in LasR-defective strains is an intriguing question that warrants further investigation. Globally, there seems to be an advantage for *P. aeruginosa* to conserve the function of LasI, while it is often not the case for LasR, as previously discussed. Finally, LasR-defective strains seem to maintain most QS-regulated functions; how this is working represents an important question to address in the coming years.

Loss of LasR function is common among *P. aeruginosa* isolated from any source, including both environmental and clinical settings, regardless of the acute or chronic nature of the infections. Furthermore, despite the absence of LasR activity, the *rhl* system is still functional in a subset of LasR-defective strains, ensuring QS functionality and the expression of survival and virulence factors. Nevertheless, it appears to be beneficial to have both LasR-defective and LasR-functional strains present in a population, as this combination of genotypes may play a beneficial role in maintaining *P. aeruginosa*’s ecological balance.

Based on available knowledge, current hypotheses, and accumulating data, we propose that LasR-defective strains isolated from infection-related settings could be acquired directly from the environment, where genomic diversification occurs in *P. aeruginosa* populations based on differences in regulation in various niches. We also propose that LasR is not as essential for functional QS as it has been commonly believed and that there may be a larger variety of QS architectures in *P. aeruginosa* than previously thought. Based on the rarity of isolates with completely deficient RhlR activity, and the relatively high frequency of LasR deficiency, we suggest that RhlR plays a more central role in QS regulation than LasR. Additionally, RhlR activity depends on MvfR-dependent regulation through the expression of *pqsE* via the *pqs* operon. More investigation on the importance of this partnership, until now mainly investigated in prototypical strains, throughout a larger panel of strains is needed, given the possible importance of the *pqs* system in maintaining QS activity in *P. aeruginosa*. Accordingly, to better understand the diversity of QS, future studies should focus on *P. aeruginosa* obtained from a broader range of environments and consider the dynamics of cocultures with naturally co-isolated strains.

## SUPPLEMENTAL MATERIAL

Supplemental Tables mbio.02376-23-s0001.pdf Tables S1 and S2.

ASM does not own the copyrights to Supplemental Material that may be linked to, or accessed through, an article. The authors have granted ASM a non-exclusive, world-wide license to publish the Supplemental Material files. Please contact the corresponding author directly for reuse.

## References

[1] Crone S, Vives-Flórez M, Kvich L, Saunders AM, Malone M, Nicolaisen MH, Martínez-García E, Rojas-Acosta C, Catalina Gomez-Puerto M, Calum H, Whiteley M, Kolter R, Bjarnsholt T. 2020. The environmental occurrence of Pseudomonas aeruginosa. APMIS 128:220–231. doi:10.1111/apm.13010 31709616

[2] Vanderwoude J, Fleming D, Azimi S, Trivedi U, Rumbaugh KP, Diggle SP. 2020. The evolution of virulence in Pseudomonas aeruginosa during chronic wound infection. Proc Biol Sci 287:20202272. doi:10.1098/rspb.2020.2272 33081616 PMC7661302

[3] Morin CD, Déziel E, Gauthier J, Levesque RC, Lau GW. 2021. An organ system-based synopsis of Pseudomonas aeruginosa virulence. Virulence 12:1469–1507. doi:10.1080/21505594.2021.1926408 34180343 PMC8237970

[4] Groleau MC, Taillefer H, Vincent AT, Constant P, Déziel E. 2022. Pseudomonas aeruginosa isolates defective in function of the LasR quorum sensing regulator are frequent in diverse environmental niches. Environ Microbiol 24:1062–1075. doi:10.1111/1462-2920.15745 34488244

[5] Schuster M, Sexton DJ, Diggle SP, Greenberg EP. 2013. Acyl-homoserine lactone quorum sensing: from evolution to application. Annu Rev Microbiol 67:43–63. doi:10.1146/annurev-micro-092412-155635 23682605

[6] Gilbert KB, Kim TH, Gupta R, Greenberg EP, Schuster M. 2009. Global position analysis of the Pseudomonas aeruginosa quorum-sensing transcription factor LasR. Mol Microbiol 73:1072–1085. doi:10.1111/j.1365-2958.2009.06832.x 19682264 PMC2759405

[7] Latifi A, Foglino M, Tanaka K, Williams P, Lazdunski A. 1996. A hierarchical quorum-sensing cascade in Pseudomonas aeruginosa links the transcriptional activators LasR and RhiR (VsmR) to expression of the stationary-phase sigma factor RpoS. Mol Microbiol 21:1137–1146. doi:10.1046/j.1365-2958.1996.00063.x 8898383

[8] Gambello MJ, Iglewski BH. 1991. Cloning and characterization of the Pseudomonas aeruginosa lasR gene, a transcriptional activator of elastase expression. J Bacteriol 173:3000–3009. doi:10.1128/jb.173.9.3000-3009.1991 1902216 PMC207884

[9] Toder DS, Gambello MJ, Iglewski BH. 1991. Pseudomonas aeruginosa LasA: a second elastase under the transcriptional control of lasR. Mol Microbiol 5:2003–2010. doi:10.1111/j.1365-2958.1991.tb00822.x 1766376

[10] Whiteley M, Lee KM, Greenberg EP. 1999. Identification of genes controlled by quorum sensing in Pseudomonas aeruginosa. Proc Natl Acad Sci U S A 96:13904–13909. doi:10.1073/pnas.96.24.13904 10570171 PMC24163

[11] Wagner VE, Bushnell D, Passador L, Brooks AI, Iglewski BH. 2003. Microarray analysis of Pseudomonas aeruginosa quorum-sensing regulons: effects of growth phase and environment. J Bacteriol 185:2080–2095. doi:10.1128/JB.185.7.2080-2095.2003 12644477 PMC151498

[12] Groleau M-C, de Oliveira Pereira T, Dekimpe V, Déziel E. 2020. PqsE is essential for RhlR-dependent quorum sensing regulation in Pseudomonas aeruginosa. mSystems 5:e00194-20. doi:10.1128/mSystems.00194-20 32457239 PMC7253366

[13] Brint JM, Ohman DE. 1995. Synthesis of multiple exoproducts in Pseudomonas aeruginosa is under the control of RhlR-RhlI, another set of regulators in strain PAO1 with homology to the autoinducer-responsive LuxR-LuxI family. J Bacteriol 177:7155–7163. doi:10.1128/jb.177.24.7155-7163.1995 8522523 PMC177595

[14] Ochsner UA, Koch AK, Fiechter A, Reiser J. 1994. Isolation and characterization of a regulatory gene affecting rhamnolipid biosurfactant synthesis in Pseudomonas aeruginosa. J Bacteriol 176:2044–2054. doi:10.1128/jb.176.7.2044-2054.1994 8144472 PMC205310

[15] Schuster M, Lostroh CP, Ogi T, Greenberg EP. 2003. Identification, timing, and signal specificity of Pseudomonas aeruginosa quorum-controlled genes: a transcriptome analysis. J Bacteriol 185:2066–2079. doi:10.1128/JB.185.7.2066-2079.2003 12644476 PMC151497

[16] Déziel E, Lépine F, Milot S, He J, Mindrinos MN, Tompkins RG, Rahme LG. 2004. Analysis of Pseudomonas aeruginosa 4-hydroxy-2-alkylquinolines (HAQs) reveals a role for 4-hydroxy-2-heptylquinoline in cell-to-cell communication. Proc Natl Acad Sci U S A 101:1339–1344. doi:10.1073/pnas.0307694100 14739337 PMC337054

[17] Xiao G, He J, Rahme LG. 2006. Mutation analysis of the Pseudomonas aeruginosa mvfR and pqsABCDE gene promoters demonstrates complex quorum-sensing circuitry. Microbiology (Reading) 152:1679–1686. doi:10.1099/mic.0.28605-0 16735731

[18] Schertzer JW, Brown SA, Whiteley M. 2010. Oxygen levels rapidly modulate Pseudomonas aeruginosa social behaviours via substrate limitation of PqsH. Mol Microbiol 77:1527–1538. doi:10.1111/j.1365-2958.2010.07303.x 20662781 PMC3098721

[19] Dulcey CE, Dekimpe V, Fauvelle DA, Milot S, Groleau MC, Doucet N, Rahme LG, Lépine F, Déziel E. 2013. The end of an old hypothesis: the Pseudomonas signaling molecules 4-hydroxy-2-alkylquinolines derive from fatty acids, not 3-ketofatty acids. Chem Biol 20:1481–1491. doi:10.1016/j.chembiol.2013.09.021 24239007 PMC3877684

[20] Cao H, Krishnan G, Goumnerov B, Tsongalis J, Tompkins R, Rahme LG. 2001. A quorum sensing-associated virulence gene of Pseudomonas aeruginosa encodes a LysR-like transcription regulator with a unique self-regulatory mechanism. Proc Natl Acad Sci U S A 98:14613–14618. doi:10.1073/pnas.251465298 11724939 PMC64730

[21] Brouwer S, Pustelny C, Ritter C, Klinkert B, Narberhaus F, Häussler S. 2014. The PqsR and RhlR transcriptional regulators determine the level of Pseudomonas quinolone signal synthesis in Pseudomonas aeruginosa by producing two different pqsABCDE mRNA isoforms. J Bacteriol 196:4163–4171. doi:10.1128/JB.02000-14 25225275 PMC4248879

[22] Dötsch A, Eckweiler D, Schniederjans M, Zimmermann A, Jensen V, Scharfe M, Geffers R, Häussler S. 2012. The Pseudomonas aeruginosa transcriptome in planktonic cultures and static biofilms using RNA sequencing. PLoS One 7:e31092. doi:10.1371/journal.pone.0031092 22319605 PMC3272035

[23] Gallagher LA, McKnight SL, Kuznetsova MS, Pesci EC, Manoil C. 2002. Functions required for extracellular quinolone signaling by Pseudomonas aeruginosa. J Bacteriol 184:6472–6480. doi:10.1128/JB.184.23.6472-6480.2002 12426334 PMC135424

[24] McGrath S, Wade DS, Pesci EC. 2004. Dueling quorum sensing systems in Pseudomonas aeruginosa control the production of the Pseudomonas quinolone signal (PQS). FEMS Microbiol Lett 230:27–34. doi:10.1016/S0378-1097(03)00849-8 14734162

[25] Drees SL, Fetzner S. 2015. PqsE of Pseudomonas aeruginosa acts as pathway-specific thioesterase in the biosynthesis of alkylquinolone signaling molecules. Chem Biol 22:611–618. doi:10.1016/j.chembiol.2015.04.012 25960261

[26] Borgert SR, Henke S, Witzgall F, Schmelz S, zur Lage S, Hotop S-K, Stephen S, Lübken D, Krüger J, Gomez NO, van Ham M, Jänsch L, Kalesse M, Pich A, Brönstrup M, Häussler S, Blankenfeldt W. 2022. Moonlighting chaperone activity of the enzyme PqsE contributes to RhlR-controlled virulence of Pseudomonas aeruginosa. Nat Commun 13:7402. doi:10.1038/s41467-022-35030-w 36456567 PMC9715718

[27] Diggle SP, Winzer K, Chhabra SR, Worrall KE, Cámara M, Williams P. 2003. The Pseudomonas aeruginosa quinolone signal molecule overcomes the cell density-dependency of the quorum sensing hierarchy, regulates rhl-dependent genes at the onset of stationary phase and can be produced in the absence of LasR. Mol Microbiol 50:29–43. doi:10.1046/j.1365-2958.2003.03672.x 14507361

[28] García-Reyes S, Cocotl-Yañez M, Soto-Aceves MP, González-Valdez A, Servín-González L, Soberón-Chávez G. 2021. PqsR-independent quorum-sensing response of Pseudomonas aeruginosa ATCC 9027 outlier-strain reveals new insights on the PqsE effect on RhlR activity. Mol Microbiol 116:1113–1123. doi:10.1111/mmi.14797 34418194

[29] Taylor IR, Paczkowski JE, Jeffrey PD, Henke BR, Smith CD, Bassler BL. 2021. Inhibitor mimetic mutations in the Pseudomonas aeruginosa PqsE enzyme reveal a protein-protein interaction with the quorum-sensing receptor RhlR that is vital for virulence factor production. ACS Chem Biol 16:740–752. doi:10.1021/acschembio.1c00049 33793200 PMC8056388

[30] Smith EE, Buckley DG, Wu Z, Saenphimmachak C, Hoffman LR, D’Argenio DA, Miller SI, Ramsey BW, Speert DP, Moskowitz SM, Burns JL, Kaul R, Olson MV. 2006. Genetic adaptation by Pseudomonas aeruginosa to the airways of cystic fibrosis patients. Proc Natl Acad Sci U S A 103:8487–8492. doi:10.1073/pnas.0602138103 16687478 PMC1482519

[31] Cabrol S, Olliver A, Pier GB, Andremont A, Ruimy R. 2003. Transcription of quorum-sensing system genes in clinical and environmental isolates of Pseudomonas aeruginosa. J Bacteriol 185:7222–7230. doi:10.1128/JB.185.24.7222-7230.2003 14645283 PMC296264

[32] D’Argenio DA, Wu M, Hoffman LR, Kulasekara HD, Déziel E, Smith EE, Nguyen H, Ernst RK, Larson Freeman TJ, Spencer DH, Brittnacher M, Hayden HS, Selgrade S, Klausen M, Goodlett DR, Burns JL, Ramsey BW, Miller SI. 2007. Growth phenotypes of Pseudomonas aeruginosa lasR mutants adapted to the airways of cystic fibrosis patients. Mol Microbiol 64:512–533. doi:10.1111/j.1365-2958.2007.05678.x 17493132 PMC2742308

[33] Hoffman LR, Kulasekara HD, Emerson J, Houston LS, Burns JL, Ramsey BW, Miller SI. 2009. Pseudomonas aeruginosa lasR mutants are associated with cystic fibrosis lung disease progression. J Cyst Fibros 8:66–70. doi:10.1016/j.jcf.2008.09.006 18974024 PMC2631641

[34] Bjarnsholt T, Jensen PØ, Fiandaca MJ, Pedersen J, Hansen CR, Andersen CB, Pressler T, Givskov M, Høiby N. 2009. Pseudomonas aeruginosa biofilms in the respiratory tract of cystic fibrosis patients. Pediatr Pulmonol 44:547–558. doi:10.1002/ppul.21011 19418571

[35] Feltner JB, Wolter DJ, Pope CE, Groleau MC, Smalley NE, Greenberg EP, Mayer-Hamblett N, Burns J, Déziel E, Hoffman LR, Dandekar AA. 2016. LasR variant cystic fibrosis isolates reveal an adaptable quorum-sensing hierarchy in Pseudomonas aeruginosa. mBio 7:e01513-16. doi:10.1128/mBio.01513-16 27703072 PMC5050340

[36] LaFayette SL, Houle D, Beaudoin T, Wojewodka G, Radzioch D, Hoffman LR, Burns JL, Dandekar AA, Smalley NE, Chandler JR, Zlosnik JE, Speert DP, Bernier J, Matouk E, Brochiero E, Rousseau S, Nguyen D. 2015. Cystic fibrosis-adapted Pseudomonas aeruginosa quorum sensing lasR mutants cause hyperinflammatory responses. Sci Adv 1:e1500199. doi:10.1126/sciadv.1500199 26457326 PMC4597794

[37] Hennemann LC, LaFayette SL, Malet JK, Bortolotti P, Yang T, McKay GA, Houle D, Radzioch D, Rousseau S, Nguyen D. 2021. LasR-deficient Pseudomonas aeruginosa variants increase airway epithelial mICAM-1 expression and enhance neutrophilic lung inflammation. PLoS Pathog 17:e1009375. doi:10.1371/journal.ppat.1009375 33690714 PMC7984618

[38] Hammond JH, Hebert WP, Naimie A, Ray K, Van Gelder RD, DiGiandomenico A, Lalitha P, Srinivasan M, Acharya NR, Lietman T, Hogan DA, Zegans ME, Blokesch M. 2016. Environmentally endemic Pseudomonas aeruginosa strains with mutations in lasR are associated with increased disease severity in corneal ulcers. mSphere 1:e00140-16. doi:10.1128/mSphere.00140-16 27631025 PMC5014915

[39] Zhao KL, Yang XT, Zeng QL, Zhang YG, Li HY, Yan CC, Li JS, Liu H, Du LM, Wu Y, Huang G, Huang T, Zhang YM, Zhou H, Wang XR, Chu YW, Zhou XK. 2023. Evolution of lasR mutants in polymorphic Pseudomonas aeruginosa populations facilitates chronic infection of the lung. Nat Commun 14:5976. doi:10.1038/s41467-023-41704-w 37749088 PMC10519970

[40] Köhler T, Buckling A, van Delden C. 2009. Cooperation and virulence of clinical Pseudomonas aeruginosa populations. Proc Natl Acad Sci U S A 106:6339–6344. doi:10.1073/pnas.0811741106 19332772 PMC2669332

[41] O’Connor K, Zhao CY, Mei M, Diggle SP. 2022. Frequency of quorum-sensing mutations in Pseudomonas aeruginosa strains isolated from different environments. Microbiology (Reading) 168:001265. doi:10.1099/mic.0.001265 36748632 PMC10233726

[42] Jeske A, Arce-Rodriguez A, Thöming JG, Tomasch J, Häussler S. 2022. Evolution of biofilm-adapted gene expression profiles in lasR-deficient clinical Pseudomonas aeruginosa isolates. NPJ Biofilms Microbiomes 8:6. doi:10.1038/s41522-022-00268-1 35165270 PMC8844440

[43] Freschi L, Jeukens J, Kukavica-Ibrulj I, Boyle B, Dupont M-J, Laroche J, Larose S, Maaroufi H, Fothergill JL, Moore M, et al.. 2015. Clinical utilization of genomics data produced by the international Pseudomonas aeruginosa consortium. Front Microbiol 6:1036. doi:10.3389/fmicb.2015.01036 26483767 PMC4586430

[44] Team RC. 2018. R: a language and environment for statistical computing. Available from: https://www.R-project.org/

[45] Mayer-Hamblett N, Rosenfeld M, Gibson RL, Ramsey BW, Kulasekara HD, Retsch-Bogart GZ, Morgan W, Wolter DJ, Pope CE, Houston LS, Kulasekara BR, Khan U, Burns JL, Miller SI, Hoffman LR. 2014. Pseudomonas aeruginosa in vitro phenotypes distinguish cystic fibrosis infection stages and outcomes. Am J Respir Crit Care Med 190:289–297. doi:10.1164/rccm.201404-0681OC 24937177 PMC4226041

[46] Mayer-Hamblett N, Ramsey BW, Kulasekara HD, Wolter DJ, Houston LS, Pope CE, Kulasekara BR, Armbruster CR, Burns JL, Retsch-Bogart G, Rosenfeld M, Gibson RL, Miller SI, Khan U, Hoffman LR. 2014. Pseudomonas aeruginosa phenotypes associated with eradication failure in children with cystic fibrosis. Clin Infect Dis 59:624–631. doi:10.1093/cid/ciu385 24863401 PMC4148602

[47] Schuster M, Greenberg EP. 2006. A network of networks: quorum-sensing gene regulation in Pseudomonas aeruginosa. Int J Med Microbiol 296:73–81. doi:10.1016/j.ijmm.2006.01.036 16476569

[48] Sandoz KM, Mitzimberg SM, Schuster M. 2007. Social cheating in Pseudomonas aeruginosa quorum sensing. Proc Natl Acad Sci U S A 104:15876–15881. doi:10.1073/pnas.0705653104 17898171 PMC2000394

[49] West SA, Griffin AS, Gardner A, Diggle SP. 2006. Social evolution theory for microorganisms. Nat Rev Microbiol 4:597–607. doi:10.1038/nrmicro1461 16845430

[50] Diggle SP, Griffin AS, Campbell GS, West SA. 2007. Cooperation and conflict in quorum-sensing bacterial populations. Nature 450:411–414. doi:10.1038/nature06279 18004383

[51] Hassett DJ, Ma JF, Elkins JG, McDermott TR, Ochsner UA, West SE, Huang CT, Fredericks J, Burnett S, Stewart PS, McFeters G, Passador L, Iglewski BH. 1999. Quorum sensing in Pseudomonas aeruginosa controls expression of catalase and superoxide dismutase genes and mediates biofilm susceptibility to hydrogen peroxide. Mol Microbiol 34:1082–1093. doi:10.1046/j.1365-2958.1999.01672.x 10594832

[52] Rumbaugh KP, Diggle SP, Watters CM, Ross-Gillespie A, Griffin AS, West SA. 2009. Quorum sensing and the social evolution of bacterial virulence. Curr Biol 19:341–345. doi:10.1016/j.cub.2009.01.050 19230668

[53] Köhler T, Buckling A, van Delden C. 2009. Cooperation and virulence of clinical Pseudomonas aeruginosa populations. Proc Natl Acad Sci U S A 106:6339–6344. doi:10.1073/pnas.0811741106 19332772 PMC2669332

[54] Harrison F, Muruli A, Higgins S, Diggle SP. 2014. Development of an ex vivo porcine lung model for studying growth, virulence, and signaling of Pseudomonas aeruginosa. Infect Immun 82:3312–3323. doi:10.1128/IAI.01554-14 24866798 PMC4136229

[55] Robitaille S, Groleau MC, Déziel E. 2020. Swarming motility growth favours the emergence of a subpopulation of Pseudomonas aeruginosa quorum-sensing mutants. Environ Microbiol 22:2892–2906. doi:10.1111/1462-2920.15042 32337826

[56] Hoffman LR, Richardson AR, Houston LS, Kulasekara HD, Martens-Habbena W, Klausen M, Burns JL, Stahl DA, Hassett DJ, Fang FC, Miller SI. 2010. Nutrient availability as a mechanism for selection of antibiotic tolerant Pseudomonas aeruginosa within the CF airway. PLoS Pathog 6:e1000712. doi:10.1371/journal.ppat.1000712 20072604 PMC2795201

[57] Barth AL, Pitt TL. 1996. The high amino-acid content of sputum from cystic fibrosis patients promotes growth of auxotrophic Pseudomonas aeruginosa. J Med Microbiol 45:110–119. doi:10.1099/00222615-45-2-110 8683546

[58] Clay ME, Hammond JH, Zhong F, Chen X, Kowalski CH, Lee AJ, Porter MS, Hampton TH, Greene CS, Pletneva EV, Hogan DA. 2020. Pseudomonas aeruginosa lasR mutant fitness in microoxia is supported by an Anr-regulated oxygen-binding hemerythrin. Proc Natl Acad Sci U S A 117:3167–3173. doi:10.1073/pnas.1917576117 31980538 PMC7022198

[59] Azimi S, Roberts AEL, Peng S, Weitz JS, McNally A, Brown SP, Diggle SP. 2020. Allelic polymorphism shapes community function in evolving Pseudomonas aeruginosa populations. ISME J 14:1929–1942. doi:10.1038/s41396-020-0652-0 32341475 PMC7368067

[60] Heurlier K, Dénervaud V, Haenni M, Guy L, Krishnapillai V, Haas D. 2005. Quorum-sensing-negative (lasR) mutants of Pseudomonas aeruginosa avoid cell lysis and death. J Bacteriol 187:4875–4883. doi:10.1128/JB.187.14.4875-4883.2005 15995202 PMC1169536

[61] Vandeplassche E, Sass A, Lemarcq A, Dandekar AA, Coenye T, Crabbé A. 2019. In vitro evolution of Pseudomonas aeruginosa AA2 biofilms in the presence of cystic fibrosis lung microbiome members. Sci Rep 9:12859. doi:10.1038/s41598-019-49371-y 31492943 PMC6731285

[62] Sousa AM, Pereira MO. 2014. Pseudomonas aeruginosa diversification during infection development in cystic fibrosis lungs-a review. Pathogens 3:680–703. doi:10.3390/pathogens3030680 25438018 PMC4243435

[63] Mould DL, Stevanovic M, Ashare A, Schultz D, Hogan DA. 2022. Metabolic basis for the evolution of a common pathogenic Pseudomonas aeruginosa variant. Elife 11:e76555. doi:10.7554/eLife.76555 35502894 PMC9224983

[64] MaClean RC, Fuentes-Hernandez A, Greig D, Hurst LD, Gudelj I. 2010. A mixture of "cheats" and "co-operators" can enable maximal group benefit. PLoS Biol 8:e1000486. doi:10.1371/journal.pbio.1000486 20856906 PMC2939026

[65] Dandekar AA, Chugani S, Greenberg EP. 2012. Bacterial quorum sensing and metabolic incentives to cooperate. Science 338:264–266. doi:10.1126/science.1227289 23066081 PMC3587168

[66] Mould DL, Botelho NJ, Hogan DA. 2020. Intraspecies signaling between common variants of Pseudomonas aeruginosa increases production of quorum-sensing-controlled virulence factors. mBio 11:e01865-20. doi:10.1128/mBio.01865-20 32843558 PMC7448281

[67] de Oliveira Pereira T, Groleau M-C, Déziel E. 2023. Surface growth of Pseudomonas aeruginosa reveals a regulatory effect of 3-oxo-C_12_-homoserine lactone in the absence of its cognate receptor, LasR. mBio 14:e0092223. doi:10.1128/mbio.00922-23 37732738 PMC10653899

[68] Williams D, Evans B, Haldenby S, Walshaw MJ, Brockhurst MA, Winstanley C, Paterson S. 2015. Divergent, coexisting Pseudomonas aeruginosa lineages in chronic cystic fibrosis lung infections. Am J Respir Crit Care Med 191:775–785. doi:10.1164/rccm.201409-1646OC 25590983 PMC4407486

[69] Hogardt M, Heesemann J. 2010. Adaptation of Pseudomonas aeruginosa during persistence in the cystic fibrosis lung. Int J Med Microbiol 300:557–562. doi:10.1016/j.ijmm.2010.08.008 20943439

[70] Ciofu O, Mandsberg LF, Bjarnsholt T, Wassermann T, Høiby N. 2010. Genetic adaptation of Pseudomonas aeruginosa during chronic lung infection of patients with cystic fibrosis: strong and weak mutators with heterogeneous genetic backgrounds emerge in mucA and/or lasR mutants. Microbiology 156:1108–1119. doi:10.1099/mic.0.033993-0 20019078

[71] Winstanley C, O’Brien S, Brockhurst MA. 2016. Pseudomonas aeruginosa evolutionary adaptation and diversification in cystic fibrosis chronic lung infections. Trends Microbiol 24:327–337. doi:10.1016/j.tim.2016.01.008 26946977 PMC4854172

[72] Fischer S, Greipel L, Klockgether J, Dorda M, Wiehlmann L, Cramer N, Tümmler B. 2017. Multilocus amplicon sequencing of Pseudomonas aeruginosa cystic fibrosis airways isolates collected prior to and after early antipseudomonal chemotherapy. J Cyst Fibros 16:346–352. doi:10.1016/j.jcf.2016.10.013 27836448

[73] Lee J, Zhang L. 2015. The hierarchy quorum sensing network in Pseudomonas aeruginosa. Protein Cell 6:26–41. doi:10.1007/s13238-014-0100-x 25249263 PMC4286720

[74] Cheng X, Lu M, Qiu H, Li Y, Huang L, Dai W. 2022. Spontaneous quorum-sensing hierarchy reprogramming in Pseudomonas aeruginosa laboratory strain PAO1. AMB Express 12:6. doi:10.1186/s13568-022-01344-7 35083573 PMC8792115

[75] Chen R, Déziel E, Groleau MC, Schaefer AL, Greenberg EP. 2019. Social cheating in a Pseudomonas aeruginosa quorum-sensing variant. Proc Natl Acad Sci U S A 116:7021–7026. doi:10.1073/pnas.1819801116 30846553 PMC6452681

[76] Asfahl KL, Smalley NE, Chang AP, Dandekar AA. 2022. Genetic and transcriptomic characteristics of RhlR-dependent quorum sensing in cystic fibrosis isolates of Pseudomonas aeruginosa. mSystems 7:e0011322. doi:10.1128/msystems.00113-22 35471121 PMC9040856

[77] Cruz RL, Asfahl KL, Van den Bossche S, Coenye T, Crabbé A, Dandekar AA. 2020. RhlR-regulated acyl-homoserine lactone quorum sensing in a cystic fibrosis isolate of Pseudomonas aeruginosa. mBio 11:e00532-20. doi:10.1128/mBio.00532-20 32265330 PMC7157775

[78] Dekimpe V, Déziel E. 2009. Revisiting the quorum-sensing hierarchy in Pseudomonas aeruginosa: the transcriptional regulator RhlR regulates LasR-specific factors. Microbiology (Reading) 155:712–723. doi:10.1099/mic.0.022764-0 19246742

[79] Kostylev M, Kim DY, Smalley NE, Salukhe I, Greenberg EP, Dandekar AA. 2019. Evolution of the Pseudomonas aeruginosa quorum-sensing hierarchy. Proc Natl Acad Sci U S A 116:7027–7032. doi:10.1073/pnas.1819796116 30850547 PMC6452656

[80] Simanek KA, Taylor IR, Richael EK, Lasek-Nesselquist E, Bassler BL, Paczkowski JE. 2022. The PqsE-RhlR interaction regulates RhlR DNA binding to control virulence factor production in Pseudomonas aeruginosa. Microbiol Spectr 10:e0210821. doi:10.1128/spectrum.02108-21 35019777 PMC8754118

[81] Letizia M, Mellini M, Fortuna A, Visca P, Imperi F, Leoni L, Rampioni G. 2022. PqsE expands and differentially modulates the RhlR quorum sensing regulon in Pseudomonas aeruginosa. Microbiol Spectr 10:e0096122. doi:10.1128/spectrum.00961-22 35604161 PMC9241726

[82] Lépine F, Milot S, Groleau MC, Déziel E. 2018. Liquid chromatography/mass spectrometry (LC/MS) for the detection and quantification of N-acyl-L-homoserine lactones (AHLs) and 4-hydroxy-2-alkylquinolines (HAQs). Methods Mol Biol 1673:49–59. doi:10.1007/978-1-4939-7309-5_4 29130163

[83] Ibrahim D, Jabbour JF, Kanj SS. 2020. Current choices of antibiotic treatment for Pseudomonas aeruginosa infections. Curr Opin Infect Dis 33:464–473. doi:10.1097/QCO.0000000000000677 33148986

[84] Fleitas Martínez O, Cardoso MH, Ribeiro SM, Franco OL. 2019. Recent advances in anti-virulence therapeutic strategies with a focus on dismantling bacterial membrane microdomains, toxin neutralization, quorum-sensing interference and biofilm inhibition. Front Cell Infect Microbiol 9:74. doi:10.3389/fcimb.2019.00074 31001485 PMC6454102

[85] Dickey SW, Cheung GYC, Otto M. 2017. Different drugs for bad bugs: antivirulence strategies in the age of antibiotic resistance. Nat Rev Drug Discov 16:457–471. doi:10.1038/nrd.2017.23 28337021 PMC11849574

[86] Lau WYV, Taylor PK, Brinkman FSL, Lee AHY. 2023. Pathogen-associated gene discovery workflows for novel antivirulence therapeutic development. EBioMedicine 88:104429. doi:10.1016/j.ebiom.2022.104429 36628845 PMC9843249

[87] Soukarieh F, Williams P, Stocks MJ, Cámara M. 2018. Pseudomonas aeruginosa quorum sensing systems as drug discovery targets: current position and future perspectives. J Med Chem 61:10385–10402. doi:10.1021/acs.jmedchem.8b00540 29999316

[88] Hentzer M, Givskov M. 2003. Pharmacological inhibition of quorum sensing for the treatment of chronic bacterial infections. J Clin Invest 112:1300–1307. doi:10.1172/JCI20074 14597754 PMC228474

[89] McInnis CE, Blackwell HE. 2011. Thiolactone modulators of quorum sensing revealed through library design and screening. Bioorg Med Chem 19:4820–4828. doi:10.1016/j.bmc.2011.06.071 21798746 PMC3152678

[90] Rutherford ST, Bassler BL. 2012. Bacterial quorum sensing: its role in virulence and possibilities for its control. Cold Spring Harb Perspect Med 2:a012427. doi:10.1101/cshperspect.a012427 23125205 PMC3543102

[91] Ahmed SAKS, Rudden M, Elias SM, Smyth TJ, Marchant R, Banat IM, Dooley JSG. 2021. Pseudomonas aeruginosa PA80 is a cystic fibrosis isolate deficient in RhlRI quorum sensing. Sci Rep 11:5729. doi:10.1038/s41598-021-85100-0 33707533 PMC7970962

[92] Roy PH, Tetu SG, Larouche A, Elbourne L, Tremblay S, Ren Q, Dodson R, Harkins D, Shay R, Watkins K, Mahamoud Y, Paulsen IT. 2010. Complete genome sequence of the multiresistant taxonomic outlier Pseudomonas aeruginosa PA7. PLoS One 5:e8842. doi:10.1371/journal.pone.0008842 20107499 PMC2809737

[93] Grosso-Becerra M-V, González-Valdez A, Granados-Martínez M-J, Morales E, Servín-González L, Méndez J-L, Delgado G, Morales-Espinosa R, Ponce-Soto G-Y, Cocotl-Yañez M, Soberón-Chávez G. 2016. Pseudomonas aeruginosa ATCC 9027 is a non-virulent strain suitable for mono-rhamnolipids production. Appl Microbiol Biotechnol 100:9995–10004. doi:10.1007/s00253-016-7789-9 27566690

