# Supplementary material for: The end of the reign of a “master regulator’’? A defect in function of the LasR quorum sensing regulator is a common feature of Pseudomonas aeruginosa isolates
Source: mBio. 2024 Feb 5;15(3):e02376-23. doi: 10.1128/mbio.02376-23 (PMC10936206; doi:10.1128/mbio.02376-23)
Supplement: Supplemental Tables — Tables S1 and S2. [file mbio.02376-23-s0001.pdf]

Table S1 : List of isolates and their origin

Isolate	Original ID	IPCD isolate ID	Origin	Human pathology	Reference
ED4334	AUS111	358	Brisbane, Australia	Urinary tract infection	Kidd *et al.* 2012 (1)
ED4451	AUS263	453	Brisbane, Australia	Urinary tract infection	Kidd *et al*. 2012 (1)
ED4336	AUS344	495	Brisbane, Australia	Urinary tract infection	Kidd *et al.* 2012 (1)
ED4450	AUS430	430	Brisbane, Australia	Urinary tract infection	Kidd *et al*. 2012 (1)
ED4337	JJ692	1177	Minneapolis, USA	Urinary tract infection	Wolfgang *et al.* 2003 (2)
ED4338	S54485	1180	Seattle, USA	Urinary tract infection	Wolfgang *et al.* 2003 (2)
DCB144-2		N/A	Ivory Coast	Urinary tract infection	This study
DCB146		N/A	Ivory Coast	Urinary tract infection	This study
DCB156		N/A	Ivory Coast	Urinary tract infection	This study
ED4339	HM293	1155	Liverpool, UK	Intestinal cancer	Freschi *et al*. 2015 (3)
ED4340	HM299	1157	Liverpool, UK	Intestinal cancer	Freschi *et al*. 2015 (3)
ED4341	HM300	1158	Liverpool, UK	Intestinal cancer	Freschi *et al*. 2015 (3)
ED4392	HM301	1159	Liverpool, UK	Intestinal cancer	Freschi *et al*. 2015 (3)
ED4342	HM306	1160	Liverpool, UK	Intestinal cancer	Freschi *et al*. 2015 (3)
ED4366-2	934436V	108	Unknown	Bronchiectasis	De Soyza *et al.* 2014 (4)
ED4367	AUS489	438	Brisbane, Australia	Bronchiectasis	Kidd *et al.* 2012 (1)
ED4368	AUS491	440	Brisbane, Australia	Bronchiectasis	Kidd *et al.* 2012 (1)
ED4369-1*	AUS496-1	441	Brisbane, Australia	Bronchiectasis	Kidd *et al.* 2012 (1)
ED4369-2*	AUS496-2	441	Brisbane, Australia	Bronchiectasis	Kidd *et al.* 2012 (1)
ED4370	AUS499	442	Brisbane, Australia	Bronchiectasis	Kidd *et al.* 2012 (1)
DCB131		N/A	Ivory Coast	Ear infection	This study
DCB132		N/A	Ivory Coast	Ear infection	This study
DCB134		N/A	Ivory Coast	Ear infection	This study
DCB135		N/A	Ivory Coast	Ear infection	This study
DCB138		N/A	Ivory Coast	Ear infection	This study
DCB139		N/A	Ivory Coast	Ear infection	This study
DCB141		N/A	Ivory Coast	Ear infection	This study
DCB142		N/A	Ivory Coast	Ear infection	This study
DCB143		N/A	Ivory Coast	Ear infection	This study
ED4381-1	AUS471	282	Brisbane, Australia	Ear infection	Kidd *et al.* 2012 (1)
ED4382	AUS134	375	Brisbane, Australia	Ear infection	Kidd *et al.* 2012 (1)
ED4383	AUS439	474	Brisbane, Australia	Ear infection	Kidd *et al.* 2012 (1)
ED4384	AUS440	475	Brisbane, Australia	Ear infection	Kidd *et al*. 2012 (1)

DCB121		N/A	Ivory Coast	Pleuropulmonary condition	This study
DCB123		N/A	Ivory Coast	Pleuropulmonary condition	This study
DCB125		N/A	Ivory Coast	Pleuropulmonary condition	This study
DCB127		N/A	Ivory Coast	Pleuropulmonary condition	This study
DCB129		N/A	Ivory Coast	Pleuropulmonary condition	This study
RM1		591	Laval, Canada	COPD	This study
ED4344	57P31PA	1274	USA	COPD	Freschi *et al*. 2015 (3)
ED4345	PA-W13	8	Nottingham, UK	Wound	Freschi *et al*. 2015 (3)
ED4346	AUS210	231	Brisbane, Australia	Wound	Kidd *et al.* 2012 (1)
ED4347	AUS407	276	Brisbane, Australia	Wound	Kidd *et al.* 2012 (1)
ED4348	So098	677	Sofia, Bulgaria	Wound	Pirnay *et al.* 2009 (5)
ED4349	A13	691	Paris, France	Wound	Pirnay *et al.* 2009 (5)
ED4350	A22	695	Paris, France	Wound	Pirnay *et al.* 2009 (5)
ED4351	PA-W31	1292	Nottingham, UK	Wound	Freschi *et al*. 2015 (3)
ED4352	PA-W39	1298	Nottingham, UK	Wound	Freschi *et al*. 2015 (3)
ED4353	PA-W42	1299	Nottingham, UK	Wound	Freschi *et al*. 2015 (3)
ED4354	PA-W46	1303	Nottingham, UK	Wound	Freschi *et al*. 2015 (3)
ED4356	PA-W8	4	Nottingham, UK	Ulcer	Freschi *et al*. 2015 (3)
ED4357	PA-W9	5	Nottingham, UK	Ulcer	Freschi *et al*. 2015 (3)
ED4358	PA-W11	6	Nottingham, UK	Ulcer	Freschi *et al*. 2015 (3)
ED4359	PA-W20	13	Nottingham, UK	Ulcer	Freschi *et al*. 2015 (3)
ED4360	A17	693	Paris, France	Leg Ulcer	Pirnay *et al.* 2009 (5)
ED4361	PA-W47	23	Nottingham, UK	Burn	Freschi *et al*. 2015 (3)
ED4362	Mi162	90	USA	Burn	Pirnay *et al.* 2009 (5)
ED4363	Lo049	852	London, UK	Burn	Pirnay *et al.* 2009 (5)
ED4364	Aa249	858	Aachen, Germany	Burn	Pirnay *et al.* 2009 (5)
ED4365	PA-W10	1284	Nottingham, UK	Burn	Freschi *et al*. 2015 (3)
ED4371	39145	109	UK	Keratitis	Stewart *et al.* 2011 (6)
ED4372	39016	1264	UK	Keratitis	Freschi *et al*. 2015 (3)
ED4373	39177	1270	Manchester, UK	Keratitis	Freschi *et al*. 2015 (3)
ED4374	152504sp2	103	Portugal	Pneumonia	Freschi *et al*. 2015 (3)
ED4375	AUS422	491	Brisbane, Australia	Pneumonia	Kidd *et al.* 2012 (1)
ED4376	AUS275	260	Brisbane, Australia	Bacteraemia	Kidd *et al.* 2012 (1)
ED4377	AUS462	266	Brisbane, Australia	Bacteraemia	Kidd *et al.* 2012 (1)
ED4378-2	AUS301-1	279	Brisbane, Australia	Bacteraemia	Kidd *et al*. 2012 (1)
ED4379	AUS150	390	Brisbane, Australia	Bacteraemia	Kidd *et al.* 2012 (1)
ED4380-1	AUS307	507	Brisbane, Australia	Bacteraemia	Kidd *et al.* 2012 (1)
ED4385	15108-1	1268	Spain	Acute infection	Freschi *et al*. 2015 (3)
ED4386-1	13121-1	1269	France	Acute infection	Freschi *et al*. 2015 (3)

ED4387	PA-W2	1	UK	Spine pressure sore	Freschi *et al*. 2015 (3)
ED4388	F2	1053	London, UK	Hyponatremia	Martin *et al.* 2013 (7)
ED4389-1	B1(P2356)	1107	Bangkok, Thailand	Primary Ciliary Diskinesia	Freschi *et al*. 2015 (3)
60	278S180511B SL_PA2	60	Montreal, Canada	Cystic Fibrosis	Freschi *et al*. 2015 (3)
84	IST27	84	Lisbon, Portugal	Cystic Fibrosis	Leitão *et al.* 1996 (8)
111	PA54A	111	Sherbrooke, Canada	Cystic Fibrosis	Freschi *et al*. 2015 (3)
147	PAC33B	147	Sherbrooke, Canada	Cystic Fibrosis	Freschi *et al*. 2015 (3)
347	AUS077	347	Brisbane, Australia	Cystic Fibrosis	Kidd *et al.* 2011 (9)
532	AUS717	532	Brisbane, Australia	Cystic Fibrosis	Kidd *et al*. 2013 (10)
549	AMT0020-84	549	Seattle, USA	Cystic Fibrosis	Freschi *et al*. 2015 (3)
700	C5311	700	Vancouver, Canada	Cystic Fibrosis	Pirnay *et al.* 2009 (5)
902	S2239	902	Dunedin, New Zealand	Cystic Fibrosis	Freschi *et al*. 2015 (3)
924	U0284	924	Hobart, Australia	Cystic Fibrosis	Freschi *et al*. 2015 (3)
1185	PA508	1185	Montreal, Canada	Cystic Fibrosis	Beaulac *et al*. 1996 (11)
1259	AA2	1259	Germany	Cystic Fibrosis	Freschi *et al*. 2015 (3)
1307	5987	1307	Québec, Canada	Cystic Fibrosis	Freschi *et al*. 2015 (3)
1357	AL6	1357	Munich, Germany	Cystic Fibrosis	Freschi *et al*. 2015 (3)
1441	VD329	1441	McMasterville, Canada	Cystic Fibrosis	Ouellet *et al.* 2014 (12)
1533	13	1533	Rovereto, Italy	Cystic Fibrosis	Freschi *et al*. 2015 (3)
1618	SMC1596	1618	Lebannon, USA	Cystic Fibrosis	(13)

N/A : Not applicable

*Two genetically different morphologies were isolated

Table S2 : Concentrations of HAQs (HHQ, PQS and HQNO), AHLS and pyocyanin quantification. All data is normalized by total protein concentration in sample.
Expression of *rhlA-gfp* reporter (RFU/OD600).

The methodology used for classification of the strains was described in Groleau *et al*., (2021) (14) and were based on a phenotypic profiling using variables contributing the most in distinguishing strains as determined by a principle components analysis (PCA). Raw data represents the relative concentration of the different extracellular metabolites (variables), namely, pyocyanin (PYO), HHQ, PQS and HQNO at two different time points (6 and 24 hours). Concentrations were normalized by the growth measured in total protein from whole cultures. Grey box indicates no data. AHL concentrations were only measured for HAQ-negative strains, and the activity of the *rhlA*-GFP reporter was only measured for LasR-defective strains in order to classify strains with RhlR Activity Independent of LasR (RAIL), as shown in Figure 2. Final classification was generated from an unbiased clustering analysis elaborated from the data obtained for the chosen variables. PA14, its *lasR* mutant and an identified RAIL strain (E90) were included in the analysis. Details on the statistical analyses are in the reference.

Strains	PYO_6h	PYO_24h	HHQ_6h	HHQ_24h	PQS_6h	PQS_24h	HQNO_6h	HQNO_24h	3-oxo-C_{12}-HSL_6h	C_4-HSL_6h	*rhlA*-GFP	Final classification
ED4334	0.010	0.027	2.987	9.751	0.501	6.592	0.959	1.237				Functional LasR
ED4451	0.128	0.228	1.456	0.614	0.152	0.994	0.331	0.322				Functional LasR
ED4336	0.227	1.075	15.479	14.096	0.450	7.353	0.562	0.807				Functional LasR
ED4450	0.038	0.056	1.269	0.089	0.230	0.902	0.189	0.169				Functional LasR
ED4337	0.024	0.108	0.213	5.987	0.211	0.478	0.025	0.825			443.547	LasR-defective
ED4338	0.017	0.058	1.670	3.230	0.303	5.587	0.234	1.378				Functional LasR
DCB144-2	0.030	0.029	0.897	7.956	0.027	0.483	0.060	0.277				LasR-defective
DCB146	0.056	0.963	0.006	0.028	0.015	0.143	0.001	0.000	0.003	0.064		LasR-defective, HAQ-negative
DCB156	0.017	0.305	1.760	15.028	0.025	3.229	0.016	0.122				LasR-defective
ED4339	0.091	0.083	2.257	0.993	0.360	1.355	0.478	0.469				Functional LasR
ED4340	0.057	0.189	1.052	0.989	0.268	0.974	0.181	0.435				Functional LasR
ED4341	0.052	0.118	1.437	1.239	0.119	1.844	0.287	0.519				Functional LasR
ED4392	0.086	0.157	1.374	1.924	0.099	3.937	0.144	0.524				Functional LasR
ED4342	0.095	0.060	0.794	0.064	0.281	1.064	0.229	0.207				Functional LasR
ED4366-2	0.031	0.224	1.427	1.078	0.214	2.656	0.084	0.471				Functional LasR
ED4367	0.036	0.019	1.090	0.030	0.442	0.334	0.284	0.047				Functional LasR
ED4368	0.048	0.213	1.093	1.811	0.315	6.338	0.145	0.366				Functional LasR
ED4369-1	0.023	0.273	0.322	2.687	0.057	0.711	0.038	0.424			1865.114	LasR-defective, RhlR-active
ED4369-2	0.080	0.078	1.841	0.274	0.058	1.356	0.116	0.210				Functional LasR
ED4370	0.071	0.130	2.437	0.485	0.255	1.737	0.356	0.312				Functional LasR
DCB131	0.077	0.785	2.488	11.881	0.055	2.659	0.141	0.594			795.692	LasR-defective
DCB132	0.156	0.855	4.022	2.710	0.064	2.346	0.275	0.472				Functional LasR
DCB134	0.096	0.437	1.976	3.010	0.163	2.470	0.321	0.758				Functional LasR
DCB135	0.056	0.057	1.556	0.162	0.361	0.640	0.093	0.075				Functional LasR
DCB138	0.083	0.065	1.509	0.118	0.208	1.076	0.123	0.209				Functional LasR
DCB139	0.031	0.792	0.003	0.014	0.009	0.154	0.000	0.001	0.002	0.039		LasR-defective, HAQ-negative
DCB141	0.090	0.054	1.510	0.099	0.524	1.047	0.171	0.120				Functional LasR
DCB142	0.059	0.553	2.377	7.133	0.014	2.032	0.080	0.649			4095.298	LasR-defective, RhlR-active
DCB143	0.077	0.191	1.518	0.116	0.212	0.772	0.180	0.177				Functional LasR
ED4381-1	0.042	0.105	1.175	0.222	0.589	3.401	0.062	0.063				Functional LasR
ED4382	0.056	0.024	0.695	12.888	0.423	0.124	0.031	0.718				LasR-defective
ED4383	0.049	0.051	2.106	0.395	0.442	3.810	0.184	0.116				Functional LasR
ED4384	0.069	0.037	3.831	26.349	0.067	0.811	0.203	0.754			1856.629	LasR-defective

DCB121	0.048	0.040	1.044	0.151	0.138	0.901	0.222	0.121				Functional LasR
DCB123	0.062	0.036	0.022	0.010	0.113	0.062	0.000	0.001	0.000	0.000		LasR-defective, HAQ-negative
DCB125	0.049	0.043	0.007	0.024	0.081	0.138	0.001	0.000	0.147	0.088		Functional LasR, HAQ-negative
DCB127	0.075	0.100	2.075	0.373	0.222	0.657	0.186	0.253				Functional LasR
DCB129	0.091	0.509	3.729	10.960	0.027	2.033	0.197	0.588			768.2022	LasR-defective
RM1-31	0.025	0.088	0.896	3.466	0.108	3.357	0.053	0.415				Functional LasR
ED4344	0.059	0.156	0.003	0.000	0.013	0.047	0.000	0.000	0.131	0.105		Functional LasR, HAQ-negative
ED4345	0.023	0.062	1.107	0.523	0.147	3.740	0.051	0.157				Functional LasR
ED4346	0.092	0.159	1.215	0.203	0.524	2.030	0.120	0.172				Functional LasR
ED4347	0.042	0.027	0.005	0.013	0.026	0.248	0.000	0.001	0.197	0.102		Functional LasR, HAQ-negative
ED4348	0.046	0.025	2.762	1.344	0.334	1.712	0.277	0.338				Functional LasR
ED4349	0.025	0.254	2.876	15.104	0.037	1.318	0.236	0.407			3460.466	LasR-defective, RhlR-active
ED4350	0.074	0.319	0.003	0.002	0.006	0.038	0.000	0.000	0.192	0.057		Functional LasR, HAQ-negative
ED4351	0.024	0.567	2.921	7.788	0.025	4.237	0.103	0.369			2321.154	LasR-defective, RhlR-active
ED4352	0.058	0.733	1.534	10.486	0.021	6.161	0.052	0.559			3143.739	LasR-defective, RhlR-active
ED4353	0.029	0.608	3.302	6.647	0.056	5.668	0.100	0.419			4460.897	LasR-defective, RhlR-active
ED4354	0.108	0.943	0.207	3.968	0.017	5.734	0.005	0.123				Functional LasR
ED4356	0.057	1.579	1.270	12.463	0.040	4.469	0.062	0.497			908.556	LasR-defective
ED4357	0.051	0.102	1.441	0.321	0.274	4.608	0.177	0.260				Functional LasR
ED4358	0.100	0.084	1.149	0.166	0.273	0.433	0.203	0.111				Functional LasR
ED4359	0.054	0.116	1.067	5.268	0.066	1.400	0.081	0.885			2551.094	LasR-defective, RhlR-active
ED4360	0.016	0.389	0.004	0.005	0.056	0.104	0.000	0.000	0.001	0.000		LasR-defective, HAQ-negative
ED4361	0.072	0.318	1.824	0.923	0.665	3.691	0.211	0.962				Functional LasR
ED4362	0.054	0.119	0.066	5.414	0.016	0.315	0.001	0.026				LasR-defective
ED4363	0.129	0.422	1.625	13.163	0.053	1.514	0.067	0.443				LasR-defective
ED4364	0.133	0.149	3.394	0.785	0.348	2.295	0.304	0.409				Functional LasR
ED4365	0.042	0.032	0.525	8.554	0.032	0.182	0.009	0.306				LasR-defective
ED4371	0.050	0.082	0.997	0.376	0.552	2.353	0.410	0.420				Functional LasR
ED4372	0.071	0.057	0.951	0.021	0.278	0.230	0.217	0.058				Functional LasR
ED4373	0.221	0.775	7.702	5.280	0.286	5.238	0.544	0.771				Functional LasR
ED4374	0.079	0.959	1.416	12.892	0.094	4.675	0.046	0.450			9145.778	LasR-defective, RhlR-active
ED4375	0.053	0.157	2.148	0.450	0.323	0.402	0.046	0.062				Functional LasR
ED4376	0.027	0.046	2.944	20.263	0.255	6.950	0.459	1.538			877.858	LasR-defective
ED4377	0.049	0.051	2.106	0.395	0.442	3.810	0.184	0.116				Functional LasR

ED4378-2	0.049	0.034	1.779	1.440	0.325	4.926	0.099	0.124				Functional LasR
ED4379	0.051	0.051	0.285	30.005	0.026	0.668	0.004	0.080				LasR-defective
ED4380-1	0.230	0.101	0.836	0.653	0.520	1.098	0.413	0.593				Functional LasR
ED4385	0.011	0.066	0.005	0.014	0.241	0.031	0.000	0.000	0.189	0.079		Functional LasR, HAQ-negative
ED4386-1	0.015	0.014	0.545	0.518	0.117	3.678	0.102	0.181				Functional LasR
ED4387	0.043	0.151	1.226	0.084	0.227	0.744	0.121	0.118				Functional LasR
ED4388	0.038	0.190	0.693	13.862	0.061	0.554	0.002	0.282			1947.333	LasR-defective, RhlR-active
ED4389-1	0.115	0.070	1.058	0.151	0.332	0.766	0.108	0.173				Functional LasR
60	0.090	0.251	0.101	0.017	0.154	0.098	0.124	0.147				Functional LasR
84	0.084	0.096	0.211	0.802	0.679	1.192	0.606	0.212				Functional LasR
111	0.009	0.065	0.008	0.010	0.063	0.057	0.001	0.001	0.000	0.001		LasR-defective, HAQ-negative
147	0.019	0.023	0.006	0.006	0.034	0.024	0.001	0.001	0.000	0.002		LasR-defective, HAQ-negative
347	0.035	0.025	16.667	35.302	0.030	0.073	0.653	2.299				LasR-defective
532	0.109	0.508	1.699	1.393	0.307	1.327	2.079	5.452				Functional LasR
549	0.405	1.888	54.543	12.628	0.562	7.254	1.458	2.144				Functional LasR
700	0.030	0.106	11.589	1.691	0.771	2.306	0.376	0.516				Functional LasR
902	0.190	1.047	2.626	0.964	0.212	1.422	0.214	0.597				Functional LasR
924	0.032	1.727	11.905	55.513	0.113	4.342	0.101	1.020				LasR-defective
1185	0.017	0.643	8.329	15.380	0.041	3.922	0.000	0.001			1683.102	LasR-defective
1259	0.059	0.049	1.752	0.280	0.611	1.233	0.591	0.399				Functional LasR
1307	0.138	0.038	3.361	5.134	0.153	0.730	0.453	1.305			11139.456	LasR-defective, RhlR-active
1357	0.021	1.118	2.956	6.033	0.069	1.190	0.049	0.241			5754.809	LasR-defective, RhlR-active
1441	0.101	0.446	3.304	4.704	0.453	1.058	0.327	0.514			5531.655	LasR-defective, RhlR-active
1533	0.029	0.159	0.949	0.799	0.311	4.921	0.312	0.895				Functional LasR
1618	0.163	0.125	3.991	0.481	0.778	1.430	0.540	0.680				Functional LasR
PA14	0.152	0.159	1.337	0.245	0.673	2.485	0.125	0.137	0.104	0.056		Functional LasR
PA14 *lasR*::Gm	0.057	0.615	0.858	10.037	0.077	2.028	0.013	0.132	0.005	0.025		LasR-defective
E90	0.233	2.158	2.602	6.620	0.027	5.952	0.482	2.715			3699.276	LasR-defective, RhlR-active

Supplementary references

1. Kidd TJ, Ritchie SR, Ramsay KA, Grimwood K, Bell SC, Rainey PB. *Pseudomonas aeruginosa* exhibits frequent recombination, but only a limited association between genotype and ecological setting. PLoS One. 2012;7(9):e44199.
2. Wolfgang MC, Kulasekara BR, Liang X, Boyd D, Wu K, Yang Q, et al. Conservation of genome content and virulence determinants among clinical and environmental isolates of *Pseudomonas aeruginosa*. Proc Natl Acad Sci U S A. 2003;100(14):8484-9.
3. Freschi L, Jeukens J, Kukavica-Ibrulj I, Boyle B, Dupont MJ, Laroche J, et al. Clinical utilization of genomics data produced by the international *Pseudomonas aeruginosa* consortium. Front Microbiol. 2015;6:1036.
4. De Soyza A, Perry A, Hall AJ, Sunny SS, Walton KE, Mustafa N, et al. Molecular epidemiological analysis suggests cross-infection with Pseudomonas aeruginosa is rare in non-cystic fibrosis bronchiectasis. European Respiratory Journal. 2014;43(3):900.
5. Pirnay JP, Bilocq F, Pot B, Cornelis P, Zizi M, Van Eldere J, et al. *Pseudomonas aeruginosa* population structure revisited. PLoS One. 2009;4(11):e7740.
6. Stewart RM, Wiehlmann L, Ashelford KE, Preston SJ, Frimmersdorf E, Campbell BJ, et al. Genetic characterization indicates that a specific subpopulation of Pseudomonas aeruginosa is associated with keratitis infections. J Clin Microbiol. 2011;49(3):993-1003.
7. Martin K, Baddal B, Mustafa N, Perry C, Underwood A, Constantidou C, et al. Clusters of genetically similar isolates of *Pseudomonas aeruginosa* from multiple hospitals in the UK. J Med Microbiol. 2013;62(Pt 7):988-1000.
8. Leitão JH, Alvim T, Sá-Correia I. Ribotyping of Pseudomonas aeruginosa isolates from patients and water springs and genome fingerprinting of variants concerning mucoidy. FEMS Immunol Med Microbiol. 1996;13(4):287-92.
9. Kidd TJ, Grimwood K, Ramsay KA, Rainey PB, Bell SC. Comparison of three molecular techniques for typing Pseudomonas aeruginosa isolates in sputum samples from patients with cystic fibrosis. J Clin Microbiol. 2011;49(1):263-8.
10. Kidd TJ, Ramsay KA, Hu H, Marks GB, Wainwright CE, Bye PT, et al. Shared Pseudomonas aeruginosa genotypes are common in Australian cystic fibrosis centres. Eur Respir J. 2013;41(5):1091-100.
11. Beaulac C, Clément-Major S, Hawari J, Lagacé J. Eradication of mucoid Pseudomonas aeruginosa with fluid liposome-encapsulated tobramycin in an animal model of chronic pulmonary infection. Antimicrob Agents Chemother. 1996;40(3):665-9.
12. Ouellet MM, Leduc A, Nadeau C, Barbeau J, Charette SJ. *Pseudomonas aeruginosa* isolates from dental unit waterlines can be divided in two distinct groups, including one displaying phenotypes similar to isolates from cystic fibrosis patients. Front Microbiol. 2014;5:802.
13. Yu Q, Griffin EF, Moreau-Marquis S, Schwartzman JD, Stanton BA, O'Toole GA. In vitro evaluation of tobramycin and aztreonam versus *Pseudomonas aeruginosa* biofilms on cystic fibrosis-derived human airway epithelial cells. J Antimicrob Chemother. 2012;67(11):2673-81.
14. Groleau MC, Taillefer H, Vincent AT, Constant P, Déziel E. *Pseudomonas aeruginosa* isolates defective in function of the LasR quorum sensing regulator are frequent in diverse environmental niches. Environ Microbiol. 2021;24(3):1062-75.
